# Association of coffee consumption pattern and metabolic syndrome among middle-aged and older adults: A cross-sectional study

**DOI:** 10.3389/fpubh.2023.1022616

**Published:** 2023-02-13

**Authors:** Ren Nina, Huang Lingling, Li Qiushuang, Guo Honglin, Sun Liyuan, Zhang Yuting

**Affiliations:** ^1^Internet Medical Center, Guangdong Second Provincial General Hospital, Guangzhou, China; ^2^Health Science Centre, Shenzhen University, Shenzhen, China; ^3^Health Management Center, Shenzhen University General Hospital, Shenzhen University Clinical Medical Academy, Shenzhen University, Shenzhen, China; ^4^School of Public Administration, South Central University for Nationalities, Wuhan, China

**Keywords:** coffee consumption, black coffee, instant coffee, fasting blood glucose, blood pressure

## Abstract

**Objectives:**

The association between coffee consumption and the risk of metabolic syndrome (MetS) remains inconsistent. The aim of this study was to evaluate the association between coffee intake and components of MetS.

**Method:**

A cross-sectional survey including 1,719 adults was conducted in Guangdong, China. Data on age, gender, education level, marriage status, body mass index (BMI), current smoking and drinking status and breakfast habit, coffee consumption type, and daily servings were derived based on 2-day, 24-h recall. MetS were assessed according to the International Diabetes Federation definition. Multivariable logistic regression was conducted to examine the association between the coffee consumption type, daily servings, and the components of MetS.

**Results:**

Regardless of the coffee type, compared with non-coffee consumers, coffee consumers had higher odds ratios (ORs) of the elevated fasting blood glucose (FBG) in both men [OR: 3.590; 95% confidence intervals (CI): 2.891–4.457] and women (OR: 3.464; 95% CI: 2.589–4.634). In women, the risk of elevated blood pressure (BP) was 0.553 times (OR: 0.553; 95% CI: 0.372–0.821, *P* = 0.004) for people who drank total coffee > 1 serving/day than for non-coffee drinkers.

**Conclusion:**

In conclusion, regardless of type, coffee intake is associated with an increased prevalence of FBG in both men and women, but has a protective effect on hypertension only in women.

## Introduction

Metabolic syndrome (MetS), defined as the presence of physiologically related cardiovascular risk factors, including dyslipidemia, abdominal obesity, hyperglycemia, and hypertension, is closely correlated with increased cardiovascular risk and common cancers (1–3). The prevalence of MetS has considerably increased over recent decades and is now at epidemic proportions worldwide (4, 5). Insulin resistance is a key hallmark feature of MetS and a critical risk factor for diabetes and other cardiovascular diseases (CVD) (6). Recently, accumulating epidemiological and experimental evidence point out that MetS is affected by genetic (7–9) and lifestyle factors (10, 11), including smoking, alcohol consumption, sugar-sweetened beverage consumption physical activity, and sedentary behaviors. Indeed, MetS have been inversely affected by dietary intakes, such as vegetables, fruits, red wine, and green tea (12). Therefore, experts emphasize dietary intakes for the primary interventions on MetS prevention (13, 14).

Coffee, which has antioxidant properties and a distinctive smell and taste, is now one of the world's most popular beverages (15). With the far-reaching development of industry and rapid changes in dietary lifestyles, coffee consumption has been considerably increasing in Shenzhen. The constituents in coffee, including polyphenols, antioxidant properties, caffeine, potassium, niacin, vitamin E, and magnesium, have been proposed to be beneficial for potential health. Experimental studies revealed that caffeine might protect against type 2 diabetes mellitus (T2DM) by stimulating free fatty acid and fat oxidation release from peripheral tissues, increasing metabolic rate and thermogenesis, and mobilizing glycogen in muscles (16). Therefore, epidemiologic studies reported a significant association between higher coffee consumption and decreased incidence of new-onset hypertension (17, 18), arterial stiffness (19, 20), T2DM (21), and promote weight loss (22). However, another study conducted in the Japanese setting demonstrated that certain types of coffee led to an increase in all-cause mortality (23). Also, other investigations reported that the intake of coffee with creamer or sugar was significantly associated with increased abdominal obesity (24) and risk of MetS (25).

The above inconsistent findings might be caused by different research designs, that is, some focused on the effect of daily coffee consumption volume, while others focused on the habitual coffee pattern. However, daily consumption patterns of coffee containing both quantitative and qualitative information are still lacking. For this reason, we performed a cross-sectional study to examine the association between coffee consumption patterns and MetS components among middle-aged and older adults.

## Materials and methods

### Study population

This cross-sectional survey was based on a large-scale, community-based routine health examination for the middle-aged and elderly. In total, 2,200 participants aged 40 years and above were recruited from January 2021 to March 2022 in Guangdong province. All individuals received a routine health check-up, including venous blood sampling and anthropometry. Among these, a subset of the individuals (*n* = 2,066) completed the 24-h food recall. Furthermore, we excluded individuals with a history of ischemic heart disease (*n* = 12) or stroke (*n* = 21), and those who take drugs to treat hyperlipidemia, diabetes, or hypertension (*n* = 314). Finally, 1,719 participants (800 men and 919 women) were included in the present study. The study was approved by the Ethics Committee of the Health Science Centre, Shenzhen University. All individuals signed written informed consent before participation.

### Diagnosis of mets

According to the guidance of the updated National Cholesterol Education Program Adult Treatment Panel III (NCEP ATP III) (26), individuals who met at least three of the following criteria were diagnosed with MetS: (1) waist circumference (WC) ≥ 90 cm in men, ≥ 80 cm in women; (2) systolic blood pressure (SBP) ≥ 130 mmHg or diastolic blood pressure (DBP) ≥ 85 mmHg; (3) fasting blood glucose (FBG) ≥ 5.60 mmol/L; (4) blood high-density lipoprotein cholesterol (HDL-C) level < 40 mg/dL in men, < 50 mg/dL in women; (5) blood triglyceride (TG) ≥ 1.70 mmol/L.

### Anthropometric and biochemical measurement

At the mobile physical examination centers, anthropometric variables including weight, height, and blood pressure (BP) were measured using standardized calibrated equipment under the guidance of professional medical staff. Body mass index (BMI, kg/m^2^) was calculated as weight divided by the height squared. WC was measured at the narrowest between the iliac crest and the bottom of the ribs. BP was measured by a sphygmomanometer (Yu yue, YJ100002, Jiangsu, China) on the right arm after the individual had been supine for at least 20 min, and the mean value of three times record was used. Biochemical assessment variables including FBG, HDL-C, TG, and 2 h post-load glucose (2hPG) were assessed using a semi-automated analyzer (Sysmex 100 XN-3000, Tokyo, Japan) enzymatically after fasting for at least 8 h. Furthermore, a detailed data collection process was reported elsewhere (27).

### Coffee consumption measurement

Information regarding coffee consumption was obtained based on a 2-day, 24-h recall. Individuals who drank coffee at least three times per week were described as coffee drinkers (28). The habitual coffee consumption questionnaire included habitual coffee type and daily coffee serving frequency. Black coffee was described as coffee powder or extracts without other ingredients. Coffee with creamer, milk, or sugar was defined as instant coffee. Other coffee is a collective name, covering a series of other types of coffee, excluding black coffee, and instant coffee. Based on the type of coffee they consumed, individuals were classified into the following five categories: non-coffee consumers, black coffee consumers, instant coffee consumers, other coffee consumers, and coffee consumers. If only black coffee or instant coffee was in a person's 2-day, 24-h food recall, the individual was determined as a black coffee or instant coffee consumer, respectively. Meanwhile, individuals who consumed any coffee type that appeared at least once were classified as coffee consumers. Other coffee consumers referred to participants who consumed other type's coffee.

### Demographic measurement

Demographic information of participants including age, gender, education level, marital status, current smoking and drinking status, breakfast habits, physical activity, and sitting time was collected through questionnaires. The level of education was categorized as up to junior high school, high school or secondary specialized school, and college and above. Marital status categories included unmarried, married or cohabiting, and others (divorced, separated, or widowed). Current smoking and drinking status were categorized as yes or no. Breakfast categories included none, 1–3 times/week, 4–5 times/week, and every day. Physical activity divided into four categories: < 0.5, 0.5–1, 1–2, and > 2 h/day. Sitting time divided into four categories: < 6, 6–8, 8–10, and > 10 h/day.

### Statistical analysis

All data are presented as mean (standard deviation) for continuous data and as percentages for categorical data according to the Shapiro–Wilk test of normality. Participants' demographic characteristics including age, education level, marital status, BMI, body weight status, current smoking and drinking status, breakfast habit, physical activity, sitting time, and Mets parameters according to coffee consumption type by gender, were compared using the Chi-square test for categorical variables and generalized linear model for continuous variables. A multivariate-adjust logistic regression model was conducted to explore the association between coffee consumption patterns and MetS components. We assigned the median of daily servings of coffee as a continuous variable and performed stratified analysis across coffee consumption categories. We adjusted covariates including BMI, education level, alcohol status, and physical activity for all the regression models, and the 95% confidence intervals (CIs) of odds ratios (ORs) were estimated. A two-sided *P-*value of < 0.05 was considered statistical significance, and SAS software (version 9.4) was used to conduct all analyses.

## Results

Table 1 presented the participants' demographic characteristics according to coffee consumption categories by gender. In men, the proportion of participants in high school or secondary specialized school was the largest for all coffee consumption categories (*P* < 0.05). In men, mean BMI and FBG levels were significantly higher in instant coffee consumers than in other groups (all *P* < 0.05). In women, mean BMI, SBP, and FBG levels were significantly higher in other coffee consumers than in other groups (all *P* < 0.05). In both men and women, the proportion of participants with normal weight status was the largest for all coffee consumption categories (all *P* < 0.05). In men, non-alcohol drinkers were more likely to be non-coffee consumers, while compared with non-alcohol drinkers, the proportion of alcohol consumers was higher than nondrinkers in the other three types of coffee pattern groups (all *P* < 0.05). In men, the duration of physical activity was higher in black coffee consumers than in the other three types of coffee pattern groups (*P* < 0.05).

**Table 1:** General characteristics of the cross-sectional study population according to coffee consumption pattern by gender.

**Variables**	**Men (n** = **800)**					**Women (n** = **919)**				
	**Non-Coffee Consumer (n** = **475)**	**Black-Coffee Consumer (*****n** =* **117)**	**Instant-Coffee Consumer (*****n** =* **66)**	**Other Coffee Consumer (*****n** =* **142)**	***P***	**Non-Coffee Consumer (n** = **549)**	**Black-Coffee Consumer (*****n** =* **143)**	**Instant-Coffee Consumer (*****n** =* **80)**	**Other Coffee Consumer (*****n** =* **147)**	***P***
**Age (years)**	62.34 ± 15.62	61.24 ± 15.53	63.00 ± 16.28	62.44 ± 15.95	0.881	61.32 ± 14.83	62.16 ± 14.75	61.31 ± 15.42	62.46 ± 14.32	0.813
**Educational level**										
~Junior high school	90 (18.95)	16 (13.68)	8 (12.12)	10 ( 7.04)	0.016	94 (17.12)	20 (13.99)	13 (16.25)	26 (17.69)	0.943
High school/Secondary specialized school	262 (55.16)	62 (52.99)	39 (59.09)	94 (66.20)		322 (58.65)	83 (58.04)	49 (61.25)	85 (57.82)	
College~	123 (25.89)	39 (33.33)	19 (28.79)	38 (26.76)		133 (24.23)	40 (27.97)	18 (22.50)	36 (24.49)	
**Marriage**										
Unmarried	155 (32.63)	41 (35.04)	25 (37.88)	59 (41.55)	0.233	193 (35.15)	55 (38.46)	26 (32.50)	55 (37.41)	0.359
Married/cohabiting	289 (60.84)	63 (53.85)	35 (53.03)	74 (52.11)		319 (58.11)	85 (59.44)	51 (63.75)	82 (55.78)	
Divorced/Separated/ Widowed	31 ( 6.53)	13 (11.11)	6 ( 9.09)	9 ( 6.34)		37 ( 6.74)	3 ( 2.10)	3 ( 3.75)	10 ( 6.80)	
Height (cm)	163.77 ± 8.11	164.38 ± 7.59	162.91 ± 7.96	163.96 ± 7.99	0.669	158.37 ± 6.39	157.68 ± 5.89	158.31 ± 6.08	157.80 ± 6.85	0.569
Weight (kg)	61.03 ± 11.05	63.90 ± 11.11	63.74 ± 10.41	62.08 ± 9.53	0.034	55.69 ± 10.29	56.85 ± 8.47	56.36 ± 9.22	57.47 ± 8.76	0.167
Body mass index	22.68 ± 3.37	23.59 ± 3.40	24.01 ± 3.35	23.07 ± 2.97	0.004	22.18 ± 3.82	22.89 ± 3.43	22.45 ± 3.18	23.09 ± 3.43	0.020
**Body weight status**										
Underweight	36 ( 7.58)	8 ( 6.84)	2 ( 3.03)	11 ( 7.75)	0.024	86 (15.66)	12 ( 8.39)	4 ( 5.00)	3 ( 2.05)	<0.001
Normal	234 (49.26)	45 (38.46)	23 (34.85)	61 (42.96)		265 (48.27)	66 (46.15)	44 (55.00)	86 (58.90)	
Overweight	117 (24.63)	31 (26.50)	16 (24.24)	33 (23.24)		102 (18.58)	34 (23.78)	15 (18.75)	24 (16.44)	
Obese	88 (18.53)	33 (28.21)	25 (37.88)	37 (26.06)		96 (17.49)	31 (21.68)	17 (21.25)	33 (22.60)	
**Current smoking status**										
Yes	223 (46.95)	59 (50.43)	26 (39.39)	70 (49.30)	0.500	236 (42.99)	67 (46.85)	34 (42.50)	71 (48.30)	0.612
No	252 (53.05)	58 (49.57)	40 (60.61)	72 (50.70)		313 (57.01)	76 (53.15)	46 (57.50)	76 (51.70)	
**Current alcohol status**										
Yes	216 (45.47)	69 (58.97)	37 (56.06)	74 (52.11)	0.032	247 (44.99)	71 (49.65)	35 (43.75)	72 (48.98)	0.647
No	259 (54.53)	48 (41.03)	29 (43.94)	68 (47.89)		302 (55.01)	72 (50.35)	45 (56.25)	75 (51.02)	
**Breakfast habit**										
No	24 ( 5.05)	6 ( 5.13)	1 ( 1.52)	8 ( 5.63)	0.661	29 ( 5.28)	6 ( 4.20)	3 ( 3.75)	1 ( 0.68)	0.334
1-3 times/week	146 (30.74)	46 (39.32)	20 (30.30)	44 (30.99)		166 (30.24)	44 (30.77)	27 (33.75)	45 (30.61)	
4-5 times/week	159 (33.47)	32 (27.35)	25 (37.88)	42 (29.58)		179 (32.60)	53 (37.06)	20 (25.00)	51 (34.69)	
Every day	146 (30.74)	33 (28.21)	20 (30.30)	48 (33.80)		175 (31.88)	40 (27.97)	30 (37.50)	50 (34.01)	
**Physical activity**										
<0.5 h/day	185 (38.95)	44 (37.61)	23 (34.85)	66 (46.48)	0.025	195 (35.52)	57 (39.86)	25 (31.25)	55 (37.41)	0.258
0.5-1 h/day	139 (29.26)	46 (39.32)	19 (28.79)	50 (35.21)		186 (33.88)	36 (25.17)	36 (45.00)	54 (36.73)	
1-2 h/day	96 (20.21)	19 (16.24)	18 (27.27)	13 ( 9.15)		101 (18.40)	31 (21.68)	12 (15.00)	21 (14.29)	
≥2 h/day	55 (11.58)	8 ( 6.84)	6 ( 9.09)	13 ( 9.15)		67 (12.20)	19 (13.29)	7 ( 8.75)	17 (11.56)	
**Sitting time**										
<6 h/day	127 (26.74)	31 (26.50)	14 (21.21)	32 (22.54)	0.679	134 (24.41)	40 (27.97)	17 (21.25)	37 (25.17)	0.650
6-8 h/day	182 (38.32)	50 (42.74)	28 (42.42)	48 (33.80)		205 (37.34)	48 (33.57)	33 (41.25)	51 (34.69)	
8-10 h/day	98 (20.63)	20 (17.09)	14 (21.21)	38 (26.76)		144 (26.23)	32 (22.38)	22 (27.50)	34 (23.13)	
≥10 h/day	68 (14.32)	16 (13.68)	10 (15.15)	24 (16.90)		66 (12.02)	23 (16.08)	8 (10.00)	25 (17.01)	
Systolic blood pressure (mmHg)	134.89 ± 20.89	137.80 ± 19.39	131.80 ± 20.51	135.68 ± 20.19	0.257	134.92 ± 22.24	128.54 ± 20.29	131.69 ± 22.81	135.09 ± 22.47	0.008
Diastolic blood pressure (mmHg)	86.32 ± 13.35	87.13 ± 12.80	85.58 ± 14.68	85.51 ± 11.77	0.742	85.13 ± 13.51	82.17 ± 14.12	82.96 ± 14.67	84.62 ± 13.64	0.119
Triglycerides (mmol/L)	1.64 ± 0.51	1.53 ± 0.49	1.69 ± 0.47	1.64 ± 0.50	0.141	1.65 ± 0.51	1.71 ± 0.50	1.58 ± 0.51	1.63 ± 0.51	0.288
High-density lipoprotein cholesterol (mmol/L)	1.25 ± 0.24	1.29 ± 0.30	1.21 ± 0.23	1.26 ± 0.27	0.304	1.30 ± 0.28	1.28 ± 0.24	1.29 ± 0.26	1.29 ± 0.25	0.811
Fasting blood glucose (mmol/L)	5.72 ± 0.46	6.01 ± 0.47	6.02 ± 0.45	5.99 ± 0.47	<0.001	5.73 ± 0.48	6.02 ± 0.42	6.00 ± 0.51	6.12 ± 0.53	<0.001
Blood glucose two hours after meals (mmol/L)	9.11 ± 0.64	9.10 ± 0.66	8.92 ± 0.63	9.07 ± 0.64	0.146	9.05 ± 0.68	9.09 ± 0.67	9.06 ± 0.67	9.20 ± 0.69	0.115

P values were calculated by generalized linear model for continuous variables and χ^2^ test for categorical variables. Underweight (BMI < 18.5 kg/m^2^); normal (BMI ≥ 18.5 and < 23 kg/m^2^); overweight (BMI ≥ 23 and < 25 kg/m^2^); obese (BMI ≥ 25 kg/m^2^).

Table 2 summarized the multivariable-adjusted OR and 95% CI of MetS components across the type of coffee by gender. Regardless of the coffee type, compared with non-coffee consumers, coffee consumers had higher ORs of the elevated FBG in both men (OR: 3.590; 95% CI: 2.891–4.457) and women (OR: 3.464; 95% CI: 2.589–4.634). In women, the prevalence of elevated blood pressure (OR: 0.661; 95% CI: 0.454–0.963) was significantly lower in black coffee consumers than in non-coffee consumers. The same inverse association can be also found in other types of coffee consumption.

**Table 2:** Multivariable-adjusted odds ratios and 95% CIs for metabolic components according to the type of coffee consumed (by gender).

**Variable**	**Non-coffee consumer (reference)**	**Black-coffee consumer**	**Instant-coffee consumer**	**Other coffee consumer**	**Coffee consumer**
**Men**					
**Elevated TG**					
Model 1	Ref	0.878 (0.582, 1.324)	0.782 (0.451, 1.355)	0.988 (0.674, 1.447)	0.903 (0.678, 1.203)
Model 2	1.139 (0.755, 1.717)	Ref	0.891 (0.471, 1.686)	1.125 (0.682, 1.857)	-
**Reduced HDL-C**					
Model 1	Ref	0.933 (0.706, 1.234)	1.211 (0.854, 1.716)	0.927 (0.709, 1.211)	0.984 (0.809, 1.198)
Model 2	1.071 (0.811, 1.416)	Ref	1.297 (0.861, 1.955)	0.993 (0.704, 1.401)	-
**Elevated BP**					
Model 1	Ref	0.867 (0.651, 1.155)	0.729 (0.510, 1.043)	0.984 (0.744, 1.302)	0.880 (0.717, 1.079)
Model 2	1.153 (0.865, 1.536)	Ref	0.841 (0.553, 1.278)	1.135 (0.796, 1.618)	-
**Elevated FBG**					
Model 1	Ref	3.523 (2.635, 4.709)	3.268 (2.273, 4.699)	3.827 (2.895, 5.059)	3.590 (2.891, 4.457)
Model 2	0.284 (0.212, 0.379)	Ref	0.928 (0.617, 1.396)	1.086 (0.776, 1.521)	-
**Women**					
**Elevated TG**					
Model 1	Ref	1.317 (0.795, 2.179)	0.736 (0.340, 1.591)	0.874 (0.497, 1.535)	1.009 (0.683, 1.490)
Model 2	0.760 (0.459, 1.257)	Ref	0.559 (0.238, 1.310)	0.664 (0.340, 1.297)	-
**Reduced HDL-C**					
Model 1	Ref	0.983 (0.676, 1.430)	0.932 (0.579, 1.501)	0.911 (0.628, 1.321)	0.943 (0.721, 1.233)
Model 2	1.017 (0.699, 1.478)	Ref	0.948 (0.543, 1.653)	0.926 (0.579, 1.482)	-
**Elevated BP**					
Model 1	Ref	0.661 (0.454, 0.963)	0.738 (0.456, 1.194)	0.984 (0.668, 1.448)	0.790 (0.600, 1.040)
Model 2	1.512 (1.038, 2.203)	Ref	1.116 (0.640, 1.947)	1.488 (0.924, 2.397)	-
**Elevated FBG**					
Model 1	Ref	2.987 (2.021, 4.413)	3.192 (1.962, 5.195)	4.177 (2.844, 6.134)	3.464 (2.589, 4.634)
Model 2	0.335 (0.227, 0.495)	Ref	1.069 (0.616, 1.854)	1.398 (0.880, 2.221)	-

CIs, confidence intervals; TG, triglyceride; HDL-C, HDL cholesterol; BP, blood pressure; FBG, fasting blood glucose; Model 1: Reference = non-coffee consumer. Adjusted for BMI, education level, alcohol status, Physical activity. Model 2: Reference = black-coffee consumer. Adjusted for BMI, education level, alcohol status, and physical activity.

We further conducted stratified analyses to explore multivariable-adjusted OR and 95% CI for MetS according to daily servings of coffee by gender, as presented in Table 3. In male black coffee drinkers, there was a linear trend between the increase of TG and the decrease in coffee consumption (*P* < 0.05). In addition, men who drank coffee > 1 serving/day had an increased risk of elevated FBG (OR: 4.112; 95% CI: 2.537–6.666; *P* < 0.05). The same results were also observed in men who drank black coffee (OR: 3.835; 95% CI: 2.009–7.319; *P* < 0.001) and instant coffee (OR: 3.651; 95% CI: 1.329–10.031; *P* < 0.001). In women, there was a positive correlation between coffee consumption and elevated FBG. The risk of elevated FBG in people who drink > 1 serving/day is 3.798 times higher than that in people who drink ≤ 1 serving/day (OR: 3.798; 95% CI: 2.555–5.647, *P* < 0.001), and the same results can be found in women who drink black coffee and instant coffee. The risk of elevated FBG was 3.109 times (OR: 3.109; 95% CI: 1.799–5.371, *P* < 0.001) in women who drank black coffee and 3.514 times (OR: 3.109; 95% CI: 1.503–8.218, *P* < 0.001) in women who drank instant coffee. In women, compared with non-coffee consumers, there was a negative correlation between coffee consumption and elevated BP. The risk of elevated BP was 0.553 times (OR: 0.553; 95% CI: 0.372–0.821, *P* = 0.004) for people who drank coffee > 1 serving/day than for non-coffee drinkers in total coffee. The risk of elevated BP was 0.516 times that of non-coffee drinkers (OR: 0.516; 95% CI: 0.296–0.898, *P* = 0.005) in black coffee. Among instant coffee drinkers, the risk of elevated BP was 0.276 times that of non-coffee drinkers (OR: 0.276; 95% CI: 0.112–0.68, *P* = 0.037).

**Table 3:** Multivariable-adjusted odds ratios and 95% CIs for metabolic components according to daily servings of coffee (by gender).

**Variable**	**Non-coffee consumer (reference)**		**Total coffee**					**Black-coffee**					**Instant-coffee**				
			≤ **1 Serving/day**		> **1 Serving/day**		***P***	≤ **1 Serving/day**		> **1 Serving/day**		***P***	≤ **1 Serving/day**		> **1 Serving/day**		***P***
**Men**																	
n		475		232		93			70		47			49		17	
Elevated TG	1		0.876 (0.545, 1.408)		0.486 (0.213, 1.108)		0.103	0.400 (0.152, 1.053)		0.348 (0.103, 1.174)		0.021	1.012 (0.443, 2.312)		0 (0, Inf)		0.240
Reduced HDL-C	1		1.032 (0.743, 1.435)		1.071 (0.678, 1.693)		0.751	0.770 (0.448, 1.323)		1.025 (0.551, 1.908)		0.731	1.588 (0.869, 2.901)		1.728 (0.650, 4.594)		0.084
Elevated BP	1		1.004 (0.704, 1.431)		1.182 (0.716, 1.953)		0.593	1.144 (0.638, 2.054)		1.637 (0.793, 3.378)		0.180	0.609 (0.323, 1.148)		0.777 (0.274, 2.208)		0.204
Elevated FBG	1		3.928 (2.726, 5.659)		4.112 (2.537, 6.666)		<0.001	4.937 (2.863, 8.513)		3.835 (2.009, 7.319)		<0.001	3.658 (1.925, 6.951)		3.651 (1.329, 10.031)		<0.001
**Women**																	
n		549		231		139			80		63			57		23	
Elevated TG	1		0.873 (0.543, 1.404)		1.044 (0.610, 1.786)		0.958	1.490 (0.793, 2.799)		0.917 (0.425, 1.980)		0.767	0.753 (0.305, 1.856)		0.603 (0.135, 2.698)		0.380
Reduced HDL-C	1		0.895 (0.652, 1.228)		0.934 (0.636, 1.371)		0.587	0.860 (0.526, 1.406)		1.061 (0.622, 1.812)		0.958	0.922 (0.526, 1.617)		0.886 (0.373, 2.102)		0.704
Elevated BP	1		0.839 (0.601, 1.171)		0.553 (0.372, 0.821)		0.004	0.602 (0.366, 0.992)		0.516 (0.296, 0.898)		0.005	1.134 (0.614, 2.092)		0.276 (0.112, 0.680)		0.037
Elevated FBG	1		3.316 (2.376, 4.627)		3.798 (2.555, 5.647)		<0.001	2.964 (1.807, 4.863)		3.109 (1.799, 5.371)		<0.001	3.077 (1.745, 5.425)		3.514 (1.503, 8.218)		<0.001

^a^P values across increasing categories of coffee consumption were tested by assigning the median of each category and as a continuous variable in the logistic regression model after adjusting for BMI, education level, alcohol status, Physical activity.

## Discussion

The current study examined the associations between coffee consumption patterns and MetS components among middle-aged and older adults in Guangdong. We found that both black coffee and instant coffee had positive associations with elevated FBG. Furthermore, these positive associations were robust in the stratified analyses among participants who consumed ≤ 1 vs. >1 Serving daily. In addition, according to gender-stratified analysis, regardless of the coffee type, women who drank a good amount of coffee were significantly associated with a lower prevalence of elevated BP than non-coffee consumers. Whereas, the same results could not be found in men. Our results revealed that habitual coffee drinking could prevent women from hypertension in a certain sense.

Our study suggested that most of the participants were of normal weight regardless of the coffee consumption type. But, in both men and women, compared with non-coffee consumers, coffee consumers were more likely to have a higher BMI. These findings are not in line with previous epidemiology studies. In a national wide cross-sectional study conducted in 2003–2004, coffee consumption was not significantly associated with BMI or waist circumference in either men or women (29). However, another cross-sectional study in Poland revealed that lower coffee consumption was significantly associated with a higher risk of obesity (30). The inconsistency may be caused by the differences in diet assessment measures and study population. The previous survey collected the data by the means of a validated food frequency questionnaire (FFQ) to measure coffee consumption, which may cause non-differential misclassification, leading to biased study results (31). Furthermore, the response categories of FFQ were close-ended, which may lead to an underestimation of coffee consumption (32). In the current survey, we adapted a 2-day, 24-h food recall to assess participants' habitual coffee consumption, which could avoid the mentioned above biases.

Unexpectedly, we found that compared to non-coffee consumers, participants who consumed ≤ 1 serving/day or >1 serving/day of any coffee were more likely to have increased FBG levels in both men and women. The results are not consistent with findings from previous epidemiologic surveys. In a cross-sectional prospective study in Dutch, higher coffee consumption tended to be significantly associated with a lower risk of T2DM (33). While numerous prospective cohort studies indicated the inverse relationship between habitual coffee consumption and the incidence of T2DM (34). This inconsistency could be attributed to various factors, such as coffee consumption type, dose, and other constitutional and environmental factors.

The mechanism of the association between coffee consumption and plasma glucose remains unclear yet. Caffeine, one of the main bioactive compounds in coffee, has numerous biological impacts on all aspects of human health (35). A previous experimental study indicated that short-term coffee consumption could impair glucose tolerance and reduce insulin sensitivity due to the A1 attenuating aortic dissection affected by the caffeine-blocking; however, this effect will not last long (36). Long-term coffee consumption could prevent the incidence of T2DM by affecting post-load rather than fasting glucose metabolism (37). On the other hand, the effect of caffeine on plasma glucose is determined by the glycemic index of food (38). From a genetic point of view, Robertson et al. revealed that the plasma glucose level might be affected by the plasma glucose level, such as rs762551 single-nucleotide polymorphism in the CYP1A2 gene, which can directly affect the rate of the body's metabolism of caffeine (39). In this aspect, CYP1A2 activity can be effected by numerous environmental factors, including race, gender, smoking, and drinking status. Another experimental study suggested that compared to baseline, fasting glucose concentrations were higher after consuming 1 L of coffee daily for 2 weeks, but not after 4 weeks, indicating that caffeine is substantially influenced by the development of tolerance (40). Therefore, the inconsistent results in the current study may be attributed to the ignorance of caffeine dose, and the participants were old, so as to affect the metabolism of caffeine.

In the current study, we found that the protective effect of habitual coffee drinking on BP was significant only in women. In line with this finding, Grosso et al. (41) reported that higher coffee consumption was associated with a decreased risk of hypertension appeared to be significant only in women (41). Actually, numerous epidemiological studies on the influence of coffee or caffeine on the incidence of CVD system have provided controversial and inconsistent findings. A systematic review and meta-analysis of randomized controlled clinical trials indicated that habitual coffee consumption can slightly increases SBP and DBP (42). In this regard, some previous studies reported a negative association between habitual coffee consumption and the risk of CVD (43, 44), while others revealed a positive association (45), or no significant association (46). Another recent meta-analysis revealed that BP elevations tended to be associated only with caffeine but not coffee (47). Thus, these conflicting findings may be due to the different types of brewing coffee, various confounding dietary factors, and the daily consuming amount.

Overall, the caffeine acute effects on BP are well-known, but the mechanism underlying the effect of chronic coffee consumption remains unclear (48). There is experimental evidence that an acute raise in BP due to coffee intake develops increasing tolerance, and intravenous caffeine led to a rise in muscle sympathetic activity and increased BP among both non-habitual and habitual coffee consumers, while coffee dietary consumption led to elevated BP on existed in non-habitual coffee consumers (49). This may be the reason that, compared to non-coffee consumers, habitual coffee consumers are less likely to show an average BP response after coffee intake. Moreover, phenolic, the main compound of coffee, can play a key role in regulating the cellular processes that lead to inflammatory responses (50). Oxidative stress has a great impact on the process that causes metabolism impairment and chronic conditions development, including hypertension (51). In this aspect, women have more antioxidants than men in natural differences (52), this may explain the gender difference in coffee consumption effect. From the point of view of genetics, lifestyle habits (such as drinking or smoking status) or genetics may influence the activity of enzymes so as to affect metabolize caffeine and BP levels. Taking into account all variables mentioned earlier, it may explain the significant protective effect of coffee intake for women but not men.

To the best of our knowledge, the current study is the first to discuss the association between coffee consumption patterns and MetS among middle-aged and older adults in Shenzhen. We assessed individuals' coffee consumption patterns upon 2-day, 24-h recall data, which can relatively obtain accurate information about habitual coffee consumption. Moreover, we estimated both coffee consumption type and daily serving times of each type, so as to provide not only qualitative but also quantitative information regarding coffee consumption patterns. However, several limitations should be noted. First, the causal associations between coffee consumption patterns and MetS could not be confirmed due to the cross-sectional nature. Second, we did not include actual consumption volumes, brewing method, sugar in coffee or other coffee ingredient consumption, total energy intake, and presence of caffeine were not obtained. Third, the study only included healthy residents, it may be potential for residual confounding factors and other lifestyle factors. Multilateral studies considering coffee consumption timing, volumes, frequency, ingredients, and other behavioral factors by gender are needed to address the association of coffee consumption patterns on MetS in a more expanded population.

## Conclusion

In conclusion, a significant positive association between coffee consumption patterns and elevated FBG in both men and women was found, whereas consumption was inversely associated with elevated BP only in women. Our findings reinforce the hypothesis on the possible benefits of hypertension for women. Due to methodological limitations, further research prospective studies or well-designed randomized controlled trials are needed to confirm the causal association.

## Data availability statement

The original contributions presented in the study are included in the article/supplementary material, further inquiries can be directed to the corresponding author.

## Ethics statement

The studies involving human participants were reviewed and approved by the Ethics Committee of the Health Science Centre, Shenzhen University. The patients/participants provided their written informed consent to participate in this study.

## Author contributions

RN and ZY conceived the study. RN, HL, LQ, GH, SL, and ZY collected data. RN and GH provided the recruitment resources. RN completed the original draft preparation. ZY reviewed, edited the final draft, and received the funding. All authors contributed to the article and approved the submitted version.
