# Correction: Association of coffee consumption pattern and metabolic syndrome among middle-aged and older adults: a cross-sectional study

**DOI:** 10.3389/fpubh.2025.1741699

**Published:** 2026-01-13

**Authors:** Ren Nina, Huang Lingling, Li Qiushuang, Guo Honglin, Sun Liyuan, Zhang Yuting

**Affiliations:** 1 Internet Medical Center, Guangdong Second Provincial General Hospital, Guangzhou, China; 2 Health Science Centre, Shenzhen University, Shenzhen, China; 3 Health Management Center, Shenzhen University General Hospital, Shenzhen University Clinical Medical Academy, Shenzhen University, Shenzhen, China; 4 School of Public Administration, South Central University for Nationalities, Wuhan, China

**Keywords:** coffee consumption, black coffee, instant coffee, fasting blood glucose, blood pressure

There was a mistake in the caption of Table 2 for the values of the ORs of the elevated FBG for women. We previously stated:

“Table 2 summarized the multivariable-adjusted OR and 95% CI of MetS components across the type of coffee by gender. Regardless of the coffee type, compared with non-coffee consumers, coffee consumers had higher ORs of the elevated FBG in both men (OR: 3.590; 95% CI: 2.891–4.457) and women (OR: 3.590; 95% CI: 2.891–4.457). In women, the prevalence of elevated blood pressure (OR: 0.661; 95% CI: 0.454–0.963) was significantly lower in black coffee consumers than in non-coffee consumers. The same inverse association can be also found in other types of coffee consumption.”

The corrected caption of Table 2 appears below.

“Table 2 summarized the multivariable-adjusted OR and 95% CI of MetS components across the type of coffee by gender. Regardless of the coffee type, compared with non-coffee consumers, coffee consumers had higher ORs of the elevated FBG in both men (OR: 3.590; 95% CI: 2.891–4.457) and women (OR: 3.464; 95% CI: 2.589–4.634). In women, the prevalence of elevated blood pressure (OR: 0.661; 95% CI: 0.454–0.963) was significantly lower in black coffee consumers than in non-coffee consumers. The same inverse association can be also found in other types of coffee consumption.”

In the abstract, there was a mistake in the values of the ORs of the elevated FBG for women. We previously stated:

“Regardless of the coffee type, compared with non-coffee consumers, coffee consumers had higher odds ratios (ORs) of the elevated fasting blood glucose (FBG) in both men [OR: 3.590; 95% confidence intervals (CI): 2.891–4.457] and women (OR: 3.590; 95% CI: 2.891–4.457).”

This has been corrected to read:

“Regardless of the coffee type, compared with non-coffee consumers, coffee consumers had higher odds ratios (ORs) of the elevated fasting blood glucose (FBG) in both men [OR: 3.590; 95% confidence intervals (CI): 2.891–4.457] and women (OR: 3.464; 95% CI: 2.589–4.634).”

The original version of this article has been updated.

